# Divulging a Pleiotropic Role of Succinate Receptor *SUCNR1* in Renal Cell Carcinoma Microenvironment

**DOI:** 10.3390/cancers14246064

**Published:** 2022-12-09

**Authors:** Rania Najm, Mahmood Yaseen Hachim, Richard K. Kandasamy

**Affiliations:** 1 College of Medicine, Mohammed Bin Rashid University of Medicine and Health Sciences, Dubai, United Arab Emirates; 2 Centre of Molecular Inflammation Research (CEMIR), Department of Clinical and Molecular Medicine (IKOM), Norwegian University of Science and Technology, 7491 Trondheim, Norway; 3 Department of Laboratory Medicine and Pathology, Center for Individualized Medicine, Mayo Clinic, Rochester, MN 55905, USA

**Keywords:** succinate receptor, renal cell carcinoma, KIRC, KIRP, tumor immune infiltrates, immunomodulators, microbiome

## Abstract

**Simple Summary:**

Renal cell carcinoma (RCC) is one of the most life-threatening urological neoplasms. The tumor microenvironment comprising immune cell infiltration is a key factor for treatment response and survival of RCC patients. In addition, several studies focused on the involvement of the microbiome in tumor progression via the secretion of metabolic by-products such as succinate. In this study, we have highlighted the potential role of the succinate receptor, SUCNR1, in modulating the tumor microenvironment in the RCC subtypes. Our data displayed a distinct association of SUCNR1 with the microbiome signature, tumor immune infiltrates, and immunomodulators in two different RCC subtypes. This correlation could have potentially contributed to the different survival outcomes of the RCC patients. Thus, SUCNR1 may serve as a promising prognostic factor that might help in improving therapeutic interventions.

**Abstract:**

The succinate receptor, SUCNR1, has been attributed to tumor progression, metastasis, and immune response modulation upon its activation via the oncometabolite succinate. Nonetheless, little is known about the prognostic relevance of SUCNR1 and its association with tumor immune infiltrates and microbiota in renal cell carcinoma (RCC). Herein, publicly available platforms including Human Protein Atlas, cBioPortal, TIMER2.0, and TISIDB were utilized to depict a divergent implication of SUCNR1 in the immune microenvironment of clear cell RCC (KIRC) and papillary RCC (KIRP); the two major subtypes of RCC. Our results showed that the SUCNR1 expression level was augmented in RCC compared to other solid cancers, yet with opposite survival rate predictions in RCC subtypes. Consequently, a higher expression level of SUCNR1 was associated with a good disease-specific survival rate (*p* = 5.797 × 10^−5^) in KIRC patients albeit a poor prognostic prediction in KIRP patients (*p* = 1.9282 × 10^−3^). Intriguingly, SUCNR1 was mainly correlated to immunomodulators and diverse immune infiltrates in KIRP. Additionally, the SUCNR1 was mostly associated with a repertoire of microbes including beneficial bacteria that likely influenced a better disease-specific survival rate in KIRC. Our findings illustrate a significant novel subtype-specific role of SUCNR1 in RCC which potentially modulates tumor immune infiltration and microbiome signature, hence altering the prognosis of cancer patients.

## 1. Introduction

Renal cell carcinoma (RCC) is a heterogeneous life-threatening malignancy that originates from kidney tubular epithelial cells and is the most common form of kidney cancer. RCC is prevalent among men and women and incidences have been increasing over the past decades [1,2,3]. Based on the pathological classification, RCC encompasses a diversified group of tumors having different genetic, molecular, and histologic alterations [4,5]. The three most common subtypes of RCC include clear cell RCC (KIRC), papillary RCC (KIRP), and chromophobe RCC (KICH). These malignancies constitute around 85%, 15%, and 5% of all kidney cancers, respectively [6]. Predictably, the clinical outcomes in RCC subtypes were comparably heterogeneous, which is a result of the fundamental differences in cancer biology between these cancer types [7,8,9]. Concordantly, KIRP patients without metastases were associated with better outcomes compared to KIRC; however, KIRP patients with metastases had the worst prognosis [10]. 

Succinate receptor hereafter refer to as SUCNR1 is a G-protein-coupled receptor (GPR91) related to the P2Y purinoreceptors family, and is activated by a Krebs cycle intermediate metabolite, succinate [11]. Until now, the activation of SUCNR1 has been linked to several pathologies such as proliferative ischemic retinopathy [12] and cardiomyocyte hypertrophy [13]. SUCNR1 was also involved in adipose tissue expansion by exerting an antilipolytic effect [14], and hepatic fibrosis upon stimulation of α-SMA production in stellate cells [15,16]. In the kidney, SUCNR1 has been associated with diabetic nephropathy via activating the renin–angiotensin system (RAS) [17,18,19].

Moreover, SUCNR1 is also linked to cancer and inflammatory pathologies [20,21,22,23,24]. There are a number of studies highlighting the metabolite composition of the tumor microenvironment, and how a unique immune and metabolic landscape promotes tumorigenesis [25]. For instance, in the gut, succinate is one of the bacterial by-products that cooperates in the growth of other pathogenic or beneficial bacteria [26], promoting tumorigenesis [27]. Interestingly, succinate-producing microbiota has also been described to induce type 2 immunity via SUCNR1 in the intestine [28]. Additionally, Zhang et al. addressed the association of SUCNR1 with immune infiltration in ovarian cancer and linked it to T cell exhaustion [29].

Recently, variation of immune cell infiltrates in the microenvironment of RCC subtypes has been reported [30] whereby, regulatory T cells and M2 macrophages were associated with unfavorable prognostic outcomes in KIRC and KIRP, respectively. The immunomodulators lymphocyte-activation 3 (LAG3) and cytotoxic T-lymphocyte-associated protein 4 (CTLA4) were associated with a poor outcome in KIRC, whereas programmed cell death ligand 2 (PD-L2) and indoleamine 2,3-dioxygenase (IDO1) were related to poor prognosis in KIRP [30]. Moreover, Heidler et al. displayed a plethora of microbiota with significant differences between healthy kidney tissue, and benign and malignant RCC tissue [31]. Yet, the role of SUCNR1 in immune infiltration and its complicity with RCC microbiota in RCC subtypes is still unclear. In the current study, we investigated the potential role of SUCNR1 in KIRC and KIRCP using datasets from multiple publicly available databases. Our results showed that the association of SUCNR1 expression levels with varying populations of immune cells, microbiota, and immunomodulators may have influenced the outcome of the two cancers. This is the first study linking gut microbiome composition to SUCNR1 expression in RCC. We strongly believe that these novel findings could potentially shed light on the SUCNR1 pleiotropism which might be considered a novel therapeutic strategy against RCC.

## 2. Materials and Methods

### 2.1. In Silico Analysis

The Human Protein Atlas (HPA) [32] was utilized to assess SUCNR1 expression among 17 cancer types and presented with box plots as median FPKM (number of Fragments Per Kilobase of exon per Million reads) and 25th and 75th percentiles. The generated data are from the Cancer Genome Atlas (TCGA). The immunohistochemistry data of SUCNR1 protein expression in 20 different types of tumor tissues were also retrieved from HPA (only 5 representative images displayed). The TIMER2.0 [33,34,35] platform was used to show the differential expression between normal and tumor tissues for SUCNR1 over all TCGA tumors. The data are demonstrated via box plots. The TISIDB [36] platform was used to estimate the correlation between SUCNR1 expression and the relative abundance of tumor-infiltrating immune cells and immunomodulators including immunostimulators and immunoinhibitors [37].

The cBioPortal [38,39] platform was used to study disease-specific survival prediction between two expression groups of SUCNR1 or microbial signature using the Kaplan–Meier curves. The two groups A and B of low and high expression levels, respectively, were divided according to the median of SUCNR1 mRNA expression z-scores relative to all samples (log RNA Seq V2 RSEM) or to the median of microbiome signature (log RNA Seq CPM). The platform was also utilized to detect the difference in microbiome signature between SUCNR1 groups A and B. The means of microbiota expression along with the *p*- and *q*-values were presented in Tables S1 and S2. The volcano plots were used to demonstrate the log2 ratio of mean in SUCNR1 group A over the mean in SUCNR1 group B of the mycobiome expression vs. −log 10 *p*-values. The correlation between SUCNR1 mRNA expression and microbiota signature was analyzed using GraphPad^TM^ software (GraphPad Prism version 9.4.1, GraphPad Software Inc., CA, USA). The cBioPortal platform was also used to generate Kaplan–Meier curves of high or low SUCNR1/ microbiota expression levels. Whereby clear cell and papillary RCC patients were divided into 4 expressing groups of low SUCNR1/low microbiota, high SUCNR1/low microbiota, low SUCNR1/high microbiota, and high SUCNR1/high microbiota.

### 2.2. Statistical Analysis

The distribution of low and high SUCNR1-expressing patient groups to renal tumor type and stages was assessed using the cBioPortal platform using the Chi-squared test. The Kaplan–Meier curves presenting disease-specific survival rates were estimated using cBioPortal and the logrank test. The Kaplan–Meier curves of high or low SUCNR1/microbiota expression levels were studied using GraphPad^TM^ software (GraphPad Software, LLC, version 9.4.1) and the logrank test. The correlation of SUCNR1 expression with an abundance of immune infiltrates and immunomodulators expression was evaluated with the TISIDB platform using Spearman’s correlation analysis. The differential expression of SUCNR1 between normal and tumor tissues across TCGA tumors was determined by TIMER2.0 using the Wilcoxon test. The difference in microbial expression between low and high SUCNR1-expressing groups was assessed by cBioPortal using Student’s *t*-test or Benjamini-Hochberg procedure and presented as mean ± SD. The correlation between SUCNR1 and microbial expression was analyzed via GraphPad^TM^ software (GraphPad Software, LLC, version 9.4.1) using Spearman’s correlation analysis. Statistical significance was reported as follows: * for *p*-value < 0.05; ** for *p*-value < 0.01; *** or **** for *p*-value <0.001.

## 3. Results

### 3.1. SUCNR1 Is Mostly Expressed in RCC

**Figure 1:**
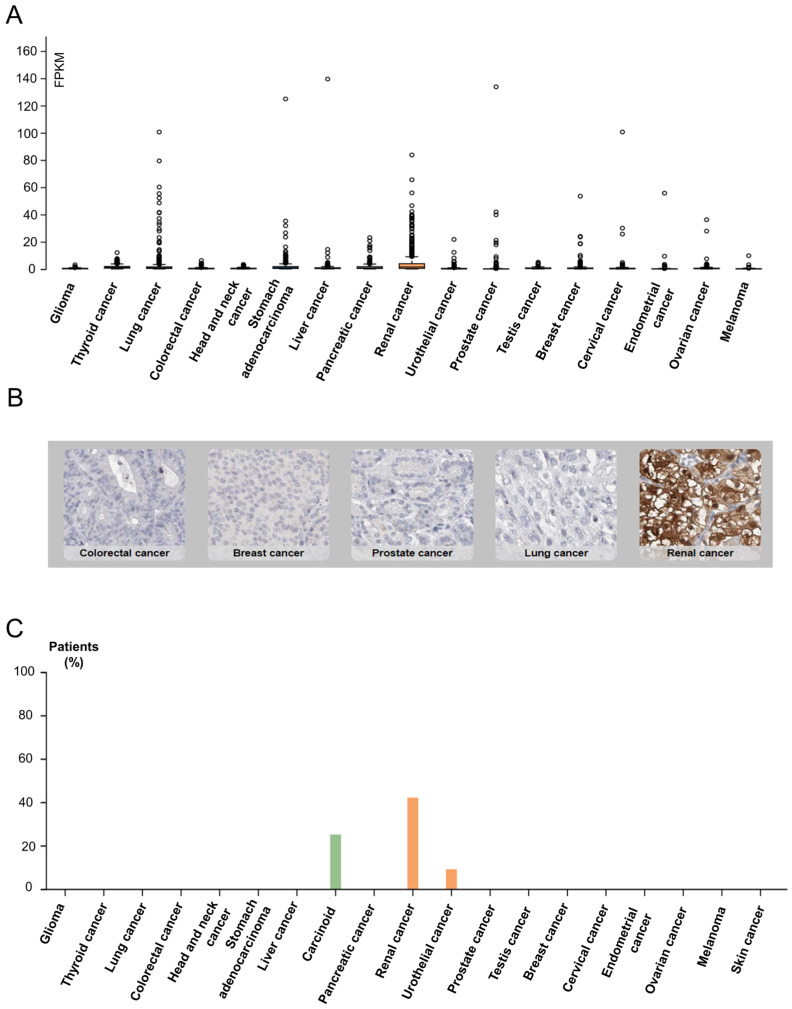
Data from Human Protein Atlas show SUCNR1 is mostly expressed in RCC. (**A**) Box plots showing the expression of SUCNR1 (FPKM) in tumor tissues of 17 different TCGA (Cancer Genome Atlas) tumors. (**B**) Immunohistochemistry showing SUCNR1 staining in colorectal, breast, prostate, lung, and renal cancer tissues. (**C**) A summary of the percentage of patients with high or medium SUCNR1 protein expression levels in 19 different cancers.

SUCNR1 is known to be mainly expressed in kidney, immune, liver, heart, and retinal cells [15]. Thus, we first inspected solid cancers for receptor expression using the Human Protein Atlas [40]. The results signify a higher SUCNR1 mRNA expression level in RCC (median = 1.4 FPKM) compared to other solid cancers such as stomach and lung cancers (median = 0.8 FPKM) (Figure 1A). This was further confirmed using data from the TIMER2.0 platform (Figure S1A). Moreover, immunohistochemistry of 20 solid tumors including a maximum of 12 patients each, shows positive SUCNR1 staining in 42% of RCC patients compared to 25% in carcinoid and 9% in urothelial cancer patients (Figure 1B,C). Although RCC had a higher SUCNR1 level compared to other cancers, both RCC tumor subtypes had lower SUCNR1 mRNA transcript compared to normal tissues (*p* < 0.001) (Figure S1B). Moreover, the SUCNR1 level in KIRC was more prominent than in KIRP (Figure S1B). This implies that SUCNR1 may utilize unique significant roles in RCC subtypes.

### 3.2. SUCNR1 Is Associated with Good Prognosis in KIRC Patients

**Figure 2:**
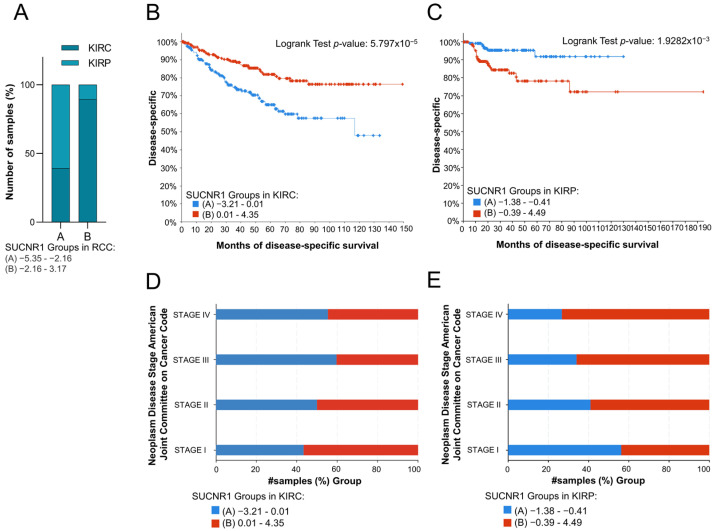
SUCNR1 is associated with good prognosis in clear cell RCC patients. (**A**) A bar graph showing the percentage of samples, divided into low expressing SUCNR1 group A or high expressing SUCNR1 group B in RCC, that belong to clear cell or papillary RCC. Chi-squared test, *p* < 10^−10^. (**B**) Kaplan–Meier curve of low expression group A and high expression group B of SUCNR1 in clear cell RCC. Logrank test, *p* = 5.797 × 10^−5^. (**C**) Kaplan–Meier curve of low expression group A and high expression group B of SUCNR1 in papillary RCC. Logrank test, *p* = 1.9282 × 10^−3^. (**D**) A bar graph showing the percentage of samples, divided into low expressing SUCNR1 group A or high expressing SUCNR1 group B in RCC, that belong to stages I, II, III, or IV of clear cell RCC. Chi-squared test, *p* = 0.0186. (**E**) A bar graph showing the percentage of samples, divided into low expressing SUCNR1 group A or high expressing SUCNR1 group B in RCC, that belong to stages I, II, III, or IV of papillary RCC. Chi-squared test, *p* = 8.932 × 10^−3^. Data available on cBioPortal platform.

To evaluate the impact of SUCNR1 on the prognosis of the RCC cancer patients, the total RCC, KIRC, and KIRP patients were divided into two groups based on the median of SUCNR1 mRNA expression level. Whereby groups A and B encompass patients with low and high SUCNR1 expression levels, respectively. The analyzed results were retrieved from the cBioPortal dataset. Interestingly, group B of total RCC patients belongs to KIRC (89.42%, *p* < 10 × 10^−10^), whereas 60.86% of group A included KIRP patients (*p* < 10 × 10^−10^) (Figure 2A). This further demonstrates that KIRC patients have higher SUCNR1 expression levels compared to KIRP. Next, we sought to investigate whether the presence of SUCNR1 has an impact on a patient’s prognosis in RCC subtypes. Group B KIRP patients, having higher expression levels of SUCNR1 were predicted with a good disease-specific survival rate (*p* = 5.797 × 10^−5^) (Figure 2B). Additionally, group KIRC B patients are mostly in stage I of cancer (56.63%, *p* = 0.018) (Figure 2D). Surprisingly, group B KIRP patients had the worst disease-specific prediction (*p* = 1.9282 × 10^−3^) (Figure 2C) leading to a higher percentage of group B KIRP patients in stage IV of cancer (73.33%, *p* = 8.932 × 10^−3^) (Figure 2E). This implies that SUCNR1 is a good prognostic factor for KIRC, unlike KIRP. Inclusively, these data highlight the major different roles that SUCNR1 played in altering the survival outcome of KIRC and KIRP patients.

### 3.3. The Expression of SUCNR1 Is Associated with a Wide Diversity of Immune Cell Subsets Infiltration in KIRP

**Figure 3:**
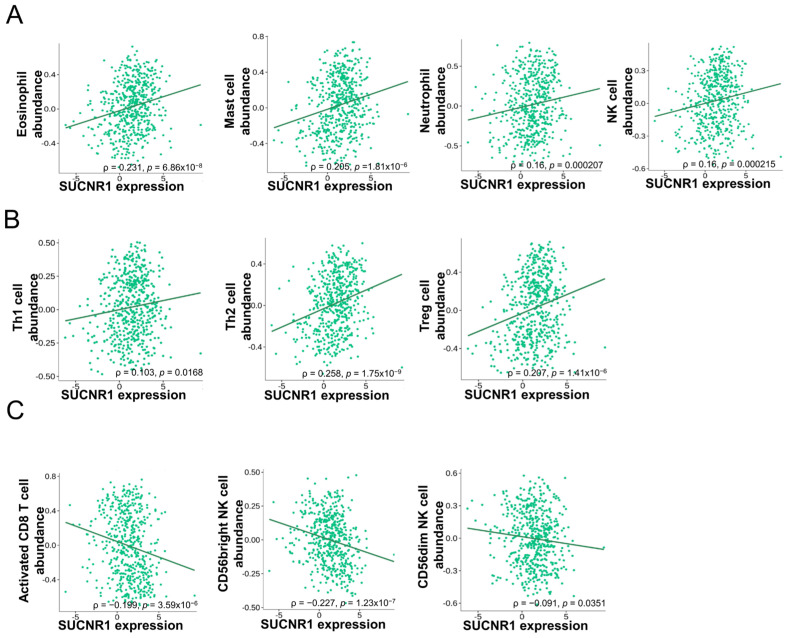
SUCNR1 expression is correlated with tumor immune infiltrates in clear cell RCC. (**A**) Positive association between SUCNR1 expression and abundance of tumor-infiltrating innate immune cells, including eosinophils, mast cells, neutrophils, and natural killer (NK) cells. (**B**) Positive correlation between SUCNR1 expression and abundance of tumor-infiltrating adaptive immune cells including T helper 1 (Th1) cells, Th2 cells, and regulatory T (Treg) cells. (**C**) Negative association between SUCNR1 expression and the abundance of tumor-infiltrating activated CD8^+^ T cells, CD56^bright^, and CD56^dim^ NK cells. Data available on TISIDB database. Spearman’s correlation coefficients (ρ) and *p*-values are displayed.

Considering the difference in function that SUCNR1 exerts in renal cancer, we questioned whether it has a distinct immune-altering role in RCC subtypes. To this end, we used the TISIDB platform to further investigate the association of SUCNR1 with tumor immune infiltration. In KIRC, the expression of SUCNR1 was positively correlated with the abundance of infiltrated innate immune cells comprising natural killer (NK) cells (ρ = 0.16, *p* = 2.15 × 10^−4^), eosinophils (ρ = 0.231, *p* = 6.86 × 10^−8^), mast cells (ρ = 0.205, *p* = 1.81 × 10^−6^), and neutrophils (ρ = 0.16, *p* = 2.7 × 10^−4^) (Figure 3A). In addition to adaptive immune cells including Th1 cells (ρ = 0.103, *p* = 0.0168), Th2 cells (ρ = 0.258, *p* = 1.75 × 10^−9^), and regulatory T cells (ρ = 0.207, *p* = 1.41 × 10^−6^) (Figure 3B). However, SUCNR1 was negatively associated with the following infiltrated immune cells activated CD8^+^ T cells (ρ = −0.199, *p* = 3.59 × 10^−6^), CD56^bright^ NK cells (ρ = −0.227, *p* = 1.23 × 10^−7^), and CD56^dim^ NK cells (ρ = −0.091, *p* = 0.0351) (Figure 3C).

**Figure 4:**
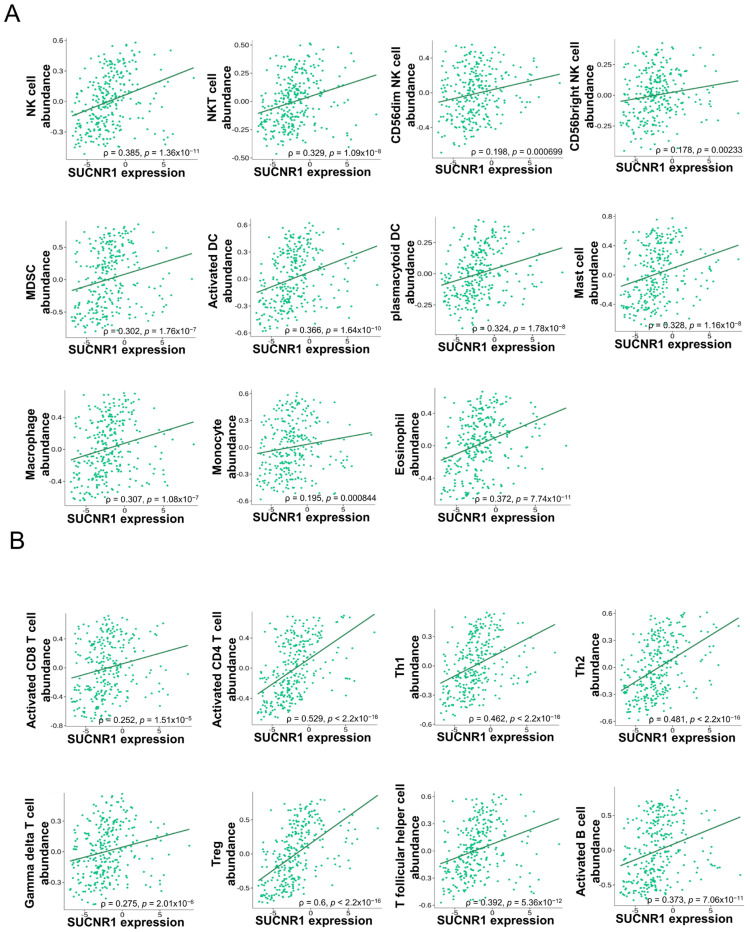
SUCNR1 expression is associated with tumor immune infiltrates in papillary RCC. (**A**) Significant association between SUCNR1 expression and abundance of tumor-infiltrating innate immune cells, including natural killer (NK) cells, NKT cells, CD56^bright^ and CD56^dim^ NK cells, myeloid-derived suppressor cells (MDSC), activated dendritic cells (DC), plasmacytoid DC, mast cells, macrophages, monocytes, and eosinophils. (**B**) Significant correlation between SUCNR1 expression and abundance of tumor-infiltrating adaptive immune cells including activated CD8^+^ T cells, activated CD4^+^ T cells, T helper 1 (Th1) cells, Th2 cells, gamma delta T cells, regulatory T (Treg) cells, T follicular helper cells, and activated B cells. Data available on TISIDB database. Spearman’s correlation coefficients (ρ) and *p*-values are displayed.

In KIRP, SUCNR1 expression was significantly correlated to the infiltration of innate immune cells, NK cells (ρ = 0.385, *p* = 1.36 × 10^−11^), NKT cells (ρ = 0.329, *p* = 1.09 × 10^−8^), CD56^bright^ NK cells (ρ = 0.178, *p* = 2.33 × 10^−3^), CD56^dim^ NK cells (ρ = 0.198, *p* = 6.9 × 10^−4^), MDSC (ρ = 0.302, *p* = 1.76 × 10^−7^), activated dendritic cells (DC) (ρ = 0.366, *p* = 1.64 × 10^−10^), plasmacytoid DC (ρ = 0.324, *p* = 1.78 × 10^−8^), mast cells (ρ = 0.328, *p* = 1.16 × 10^−8^), macrophages (ρ = 0.307, *p* = 1.06 × 10^−7^), monocytes (ρ = 0.195, *p* = 8.44 × 10^−4^), and eosinophils (ρ = 0.372, *p* = 7.74 × 10^−11^) (Figure 4A), along with adaptive immune cells such as activated CD8^+^ T cells (ρ = 0.252, *p* = 1.51 × 10^−5^), activated CD8^+^ T cells (ρ = 0.529, *p* < 2.2 × 10^−16^), Th1 cells (ρ = 0.462, *p* < 2.2 × 10^−16^), Th2 cells (ρ = 0.481, *p* < 2.2 × 10^−16^), gamma delta T cells (ρ = 0.275, *p* = 2.01 × 10^−6^), regulatory T cells (ρ = 0.6, *p* < 2.2 × 10^−16^), T follicular helper cells (ρ = 0.392, *p* = 5.36 × 10^−12^), and activated B cells (ρ = 0.373, *p* = 7.06 × 10^−11^) (Figure 4B).

Collectively, these results showed that SUCNR1 is associated with a variety of immune cell subsets in KIRP compared to KIRC. This further emphasized the divergent role that SUCNR1 specifically plays in altering the tumor immune infiltration dependent on the RCC subtype.

### 3.4. The Expression of SUCNR1 Is Correlated with a Wide Range of Immunomodulators in KIRP

**Figure 5:**
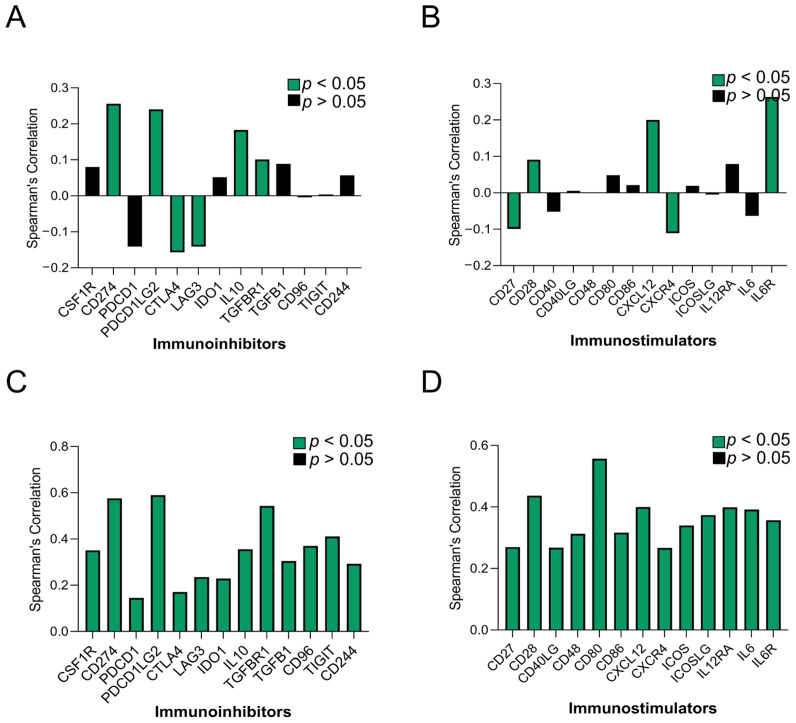
SUCNR1 expression is associated with a wide range of immunomodulators in papillary RCC. A summary of correlation between SUCNR1 expression and immunomodulators in clear cell RCC. (**A**) The immunoinhibitors include CSF1R, CD274, PDCD1, PDCD1LG2, CTLA4, LAG3, IDO1, IL10, TGFBR1, TGFB1, CD96, TIGIT, and CD244. (**B**) The immunostimulators include CD27, CD28, CD40LG, CD48, CD80, CD86, CXCL12, CXCR4, ICOS, ICOSLG, IL12RA, IL6, and IL6R. A summary of association between SUCNR1 expression and immunomodulator in papillary RCC. (**C**) The immunoinhibitors include CSF1R, CD274, PDCD1, PDCD1LG2, CTLA4, LAG3, IDO1, IL10, TGFBR1, TGFB1, CD96, TIGIT, and CD244. (**D**) The immunostimulators include CD27, CD28, CD40LG, CD48, CD80, CD86, CXCL12, CXCR4, ICOS, ICOSLG, IL12RA, IL6, and IL6R. Data available on TISIDB database. Spearman’s correlation coefficients (ρ) are displayed. *p*-values are color coded; *p* < 0.05 in green and *p* > 0.05 in black.

To elaborate more on the immune regulatory role of SUCNR1 in KIRC and KIRP, we studied the association of SUCNR1 with immunoinhibitors and immunostimulators. Using the TISIDB platform, the SUCNR1 expression level was found to be correlated to the expression level of a few immunomodulators (Figure 5A,B). This includes immuno-inhibitors such as, CD274 (ρ = 0.256, *p* = 2.2 × 10^−9^), IL10 (ρ = 0.183, *p* = 2.08 × 10^−5^), PDCD1LG2 (ρ = 0.24, *p* = 2.13 × 10^−8^), TGFBR (ρ = 0.101, *p* = 0.0195), CTLA4 (ρ = −0.157, *p* = 2.6 × 10^−4^), and LAG3 (ρ = −0.141, *p* = 0.001) (Figure 5A). Regarding the immunostimulators, SUCNR1 expression was correlated with CXCL12 (ρ = 0.202, *p* = 2.57 × 10^−6^), IL6R (ρ = 0.263, *p* = 7.87 × 10^−10^), CD28 (ρ = 0.091, *p* = 0.035), CD27 (ρ = −0.099, *p* = 0.0215), and CXCR4 (ρ = −0.111, *p* = 0.0102) (Figure 5B).

On the contrary, in KIRP, SUCNR1 expression was significantly (*p* < 0.001) associated with a wide repertoire of immunoinhibitors including; CD244 (ρ = 0.293), CD274 (ρ = 0.576), CD96 (ρ = 0.37), CSF1R (ρ = 0.351), CTLA4 (ρ = 0.171), IDO1 (ρ = 0.229), IL10 (ρ = 0.356), LAG3 (ρ = 0.235), PDCD1 (ρ = 0.145), PDCD1LG2 (ρ = 0.59), TGFB1 (ρ = 0.305), TGFBR1 (ρ = 0.543), and TIGIT (ρ = 0.411) (Figure 5C). In addition to immunostimulators such as CD27 (ρ = 0.27), CD28 (ρ = 0.437), CD40LG (ρ = 0.268), CD48 (ρ = 0.313), CD80 (ρ = 0.557), CD86 (ρ = 0.317), CXCL12 (ρ = 0.4), CXCR4 (ρ = 0.267 ICOS (ρ = 0.34), ICOSLG (ρ = 0.374), IL2RA (ρ = 0.399), IL6 (ρ = 0.392), and IL6R (ρ = 0.358) (Figure 5D).

These observations highlight the involvement of SUCNR1 in the tumor immunity of RCC subtypes, and of KIRP to more extent. Accordingly, the consequential correlation between SUCNR1 expression and diverse immunomodulators in KIRP, emphasizes the role of the receptor in immune-specific modulation and potential survival outcome.

### 3.5. SUCNR1 Is Associated with Different Microbiome Signatures in RCC Subtypes

**Figure 6:**
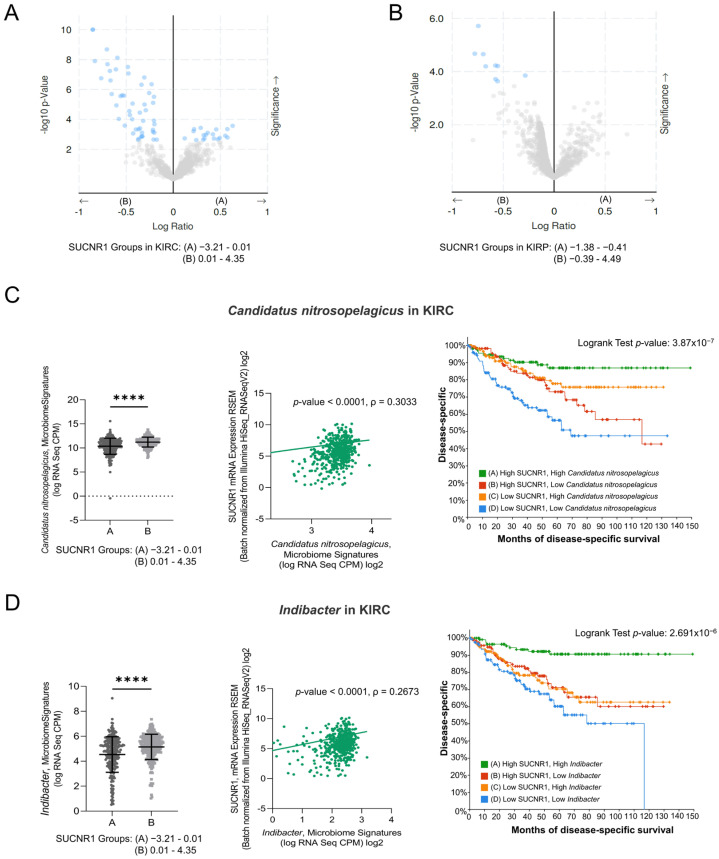
SUCNR1 is linked to a favorable microbiome signature in clear cell RCC. (**A**) The volcano plots represent the correlation between SUCNR1 expression and microbiota signature. log2 ratio of means in high expressing SUCNR1 group A to means in low expressing SUCNR1 group B of the mycobiome expression vs. −log 10 *p*-value are shown in clear cell RCC and in (**B**) papillary RCC. (**C**) Beneficial association between SUCNR1 and *Candidatus nitrosopelegicus*. Left panel: Significant increase in *Candidatus nitrosopelegicus* signature (log CPM) in high SUCNR1 expressing group B. Student’s *t*-test: **** *p* < 0.0001 B. Middle panel: Positive correlation between SUCNR1 (RSEM) expression and *Candidatus nitrosopelegicus* signature (log CPM). Spearman’s correlation coefficient, ρ = 0.3033 and *p* < 0.0001. Right panel: Kaplan–Meier curves of high or low SUCNR1/*Candidatus nitrosopelegicus*. Logrank test significant between groups; A–B (*p* = 4.09 × 10^−3^), A–D (*p* = 7.59 × 10^−7^), B–D (*p* = 5.70 × 10^−3^), C–D (*p* = 2.52 × 10^−4^). (**D**) Beneficial association between SUCNR1 and *Indibacter*. Left panel: Significant increase in *Indibacter* signature (log CPM) in high SUCNR1 expressing group B. Student’s *t*-test: **** *p* < 0.0001. Middle panel: Positive correlation between SUCNR1 (RSEM) expression and *Indibacter* signature (log CPM). Spearman’s correlation coefficient, ρ = 0.2673 and *p* < 0.0001. Right panel: Kaplan–Meier curves of high or low SUCNR1/*Indibacter.* Logrank test significant between groups; A–B (*p* = 2.04 × 10^−4^), A–C (*p* = 7.17 × 10^−5^), A–D (*p* = 4.31 × 10^−8^). Data available on cBioPortal platform.

Recently, tumor microbiota has been the focus of analyzing the function and induction of cancer patients’ immune responses. We assessed the relation between the expression of SUCNR1 and the microbiome in RCC using cBioPortal datasets. The KIRC and KIRP patients were divided into groups A and B based on low and high SUCNR1 expression levels, respectively. The significantly different microbiome present in both groups and their mean of expression (log RNA Seq CPM) are listed in Table S1 and S2. Interestingly, the results illustrated a significant positive correlation between SUCNR1 and the expression of 52 microbes in KIRC (Table S3). Among these, 19 microbes in SUCNR1 group A were negatively associated with SUCNR1 expression (Figure 6A, Table S3). In KIRP, only 9 microbes were found in group B and were positively correlated with SUCNR1 expression (Figure 6B, Table S4). Hence, SUCNR1 is related to a unique microbiome signature in each RCC subtype.

### 3.6. SUCNR1 Is Linked to a Favorable Microbiome Signature in KIRC

Interestingly, five of the significantly associated bacteria to SUCNR1 group B were common between KIRC and KIRP (Tables S1 and S2). This includes the genera *Indibacter*, *Candidatus nitrosopelagicus*, *Lachnoclostridium*, *Desulfotalea*, and *Flavonifractor*. To investigate the role of these bacteria in the prognosis of KIRC and KIRP patients, the patients were divided according to the median microbiome expression levels. Therefore, groups A and B reflect low and high levels of the corresponding bacteria, respectively. Intriguingly, only two out of the five common genera, *Candidatus nitrosopelagicus* (*p* = 4.531 × 10^−6^) and *Indibacter* (*p* = 3.533 × 10^−4^), were associated with good disease-specific survival prediction in KIRC patients (Figure S2). Yet, none of the genera had any effect on the prognosis of KIRP patients.

As such, we further evaluated the relation between *Candidatus nitrosopelagicus* and *Indibacter* with SUCNR1 in KIRC. The expression of SUCNR1 was positively correlated with *Candidatus nitrosopelagicus* (ρ = 0.3033, *p* < 0.0001) (Figure 6C), and *Indibacter* (ρ = 0.2673, *p* < 0.0001) (Figure 6D). Moreover, patients with high levels of SUCNR1 and high levels of *Candidatus nitrosopelagicus* or *Indibacter* are associated with better disease-specific survival prediction compared to patients of low SUCNR1 levels and low levels of either of the bacteria (*p* < 0.0001) (Figure 6C,D). These results demonstrate a probable fundamental function of SUCNR1 in incorporating beneficial microbiota which may lead to a better survival outcome in KIRC patients.

**Figure 7:**
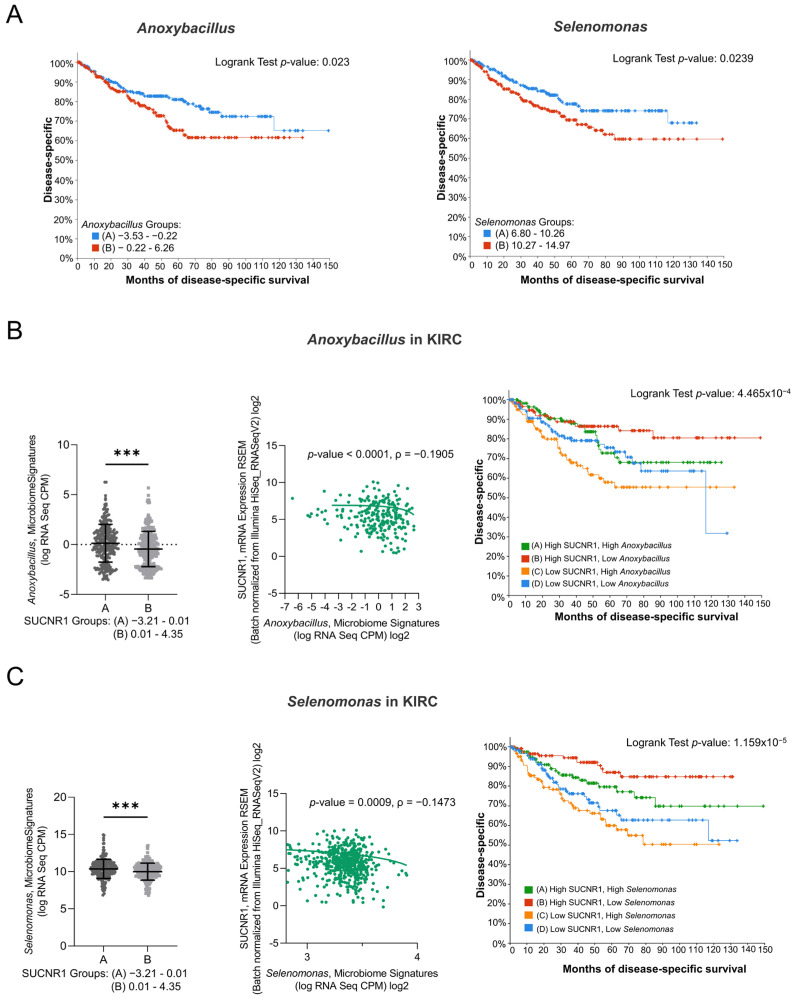
SUCNR1 negatively correlated with pathogenic microbiota in clear cell RCC. (**A**) Kaplan–Meier curves of low signature group A and high signature group B of *Anoxybacillus* (Logrank test, *p* = 0.023) and *Selenomonas* (Logrank test, *p* = 0.0239) in clear cell RCC. (**B**) Negative association between SUCNR1 and *Anoxybacillus*. Left panel: Significant increase in *Anoxybacillus* signature (log CPM) in low SUCNR1 expressing group A. Student’s *t*-test: *** *p* < 0.0001. Middle panel: Negative correlation between SUCNR1 (RSEM) expression and *Anoxybacillus* signature. Spearman’s correlation coefficient, ρ = −0.1905 and *p* < 0.0001. Right panel: Kaplan–Meier curves of high or low SUCNR1/*Anoxybacillus*. Logrank test significant between groups; A–B (*p* = 7.23 × 10^−3^), B–C (*p* = 9.67 × 10^−5^), B–D (*p* = 0.0198). (**C**) Negative association between SUCNR1 and *Selenomonas*. Left panel: Significant increase in *Indibacter* signature (log CPM) in low SUCNR1 expressing group A. Student’s *t*-test: *** *p* < 0.0001. Middle panel: Negative correlation between SUCNR1 (RSEM) expression and *Selenomonas* signature. Spearman’s correlation coefficient, ρ = −0.1473 and *p* = 0.0009. Right panel: Kaplan–Meier curves of high or low SUCNR1/*Selenomonas.* Logrank test significant between groups; A–B (*p* = 0.0395), A–C (*p* = 4.24 × 10^−3^), B–C (*p* = 1.98 × 10^−6^), B–D (*p* = 2.79 × 10^−4^). Data available on cBioPortal platform.

To emphasize the role of SUCNR1 in KIRC, the impact of the microbiome in SUCNR1 group A on patient survival was studied. Only *Anoxybacillus* and *Selenomonas genera*, which were high in SUCNR1 group A (Figure 7B,C), displayed a poor disease-specific survival prediction in KIRC patients (Figure 7A,B). Yet, the expression level of *Anoxybacillus* (ρ = −0.1905, *p* < 0.0001) and *Selenomonas* (ρ = −0.1473, *p* = 0.0009) were negatively associated with SUCNR1 expression (Figure 7B,C). Additionally, high levels of SUCNR1 and low levels of either of the bacteria showed better disease-specific survival rates (*p* < 0.001) (Figure 7B,C). Therefore, SUCNR1 expression in KIRC is a good prognostic factor and is usually associated with a favorable microbiome signature.

### 3.7. SUCNR1 Is Related to Pathogenic Microbiota in KIRP

**Figure 8:**
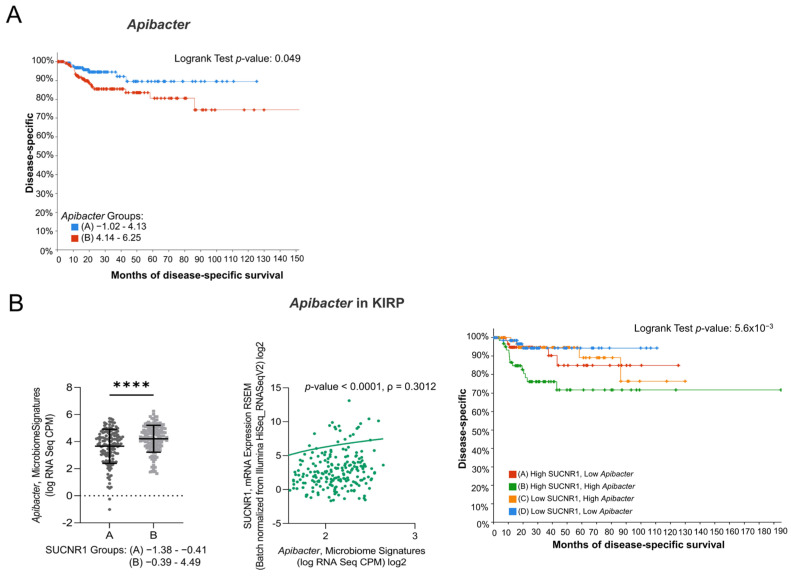
SUCNR1 is related to pathogenic microbiota in papillary RCC. (**A**) Kaplan–Meier curves of low signature group A and high signature group B of *Apibacter* in papillary RCC. Logrank test, *p* = 0.049. (**B**) Positive association between SUCNR1 and *Apibacter*. Left panel: Significant increase in *Apibacter* signature (log CPM) in high SUCNR1 expressing group B. Student’s *t*-test: **** *p* < 0.0001. Middle panel: Positive correlation between SUCNR1 (RSEM) expression and *Apibacter* signature. Spearman’s correlation coefficient, ρ = 0.3012 and *p* < 0.0001. Right panel: Kaplan–Meier curves of high or low SUCNR1/ *Apibacter* in papillary RCC. Logrank test significant between groups; A–B (*p* = 0.0413), B–D (*p* = 3.91 × 10^−3^). Data available on cBioPortal platform.

Since the SUCNR1-related microbiome in KIRC elucidated a positive outcome of the receptor, we explored the microbiome signature in KIRP. Only one genus, *Apibacter*, that was high in SUCNR1 group B (Figure 8B) showed a bad prognosis (*p* = 0.049) (Figure 8A). Moreover, SUCNR1 expression was significantly correlated with the expression of *Apibacter* (ρ = 0.3012, *p* < 0.0001) (Figure 8B). Patients with high levels of SUCNR1 and high levels of *Apibacter* are associated with the worst disease-specific survival prognosis compared to patients with low SUCNR1 levels and low levels of the bacteria (*p* = 3.91 × 10^−3^) (Figure 8B). As such, the poor survival outcome of KIRP patients with high SUCNR1 expression might be explained by the presence of pathogenic microbes.

## 4. Discussion

The multifaceted receptor, SUCNR1, has been attributed to kidney physiopathology via stimulation of the local and systemic renin–angiotensin system, development of metabolic syndrome, and hypertension [41,42]. However, its role in RCC subtypes, encompassing patient prognosis, tumor immune infiltration, and microbiome signature, is yet to be determined. In this study, we described a favorable outcome of SUCNR1 in KIRC patients contrary to its attribution in KIRP. The expression of SUCNR1 predicted a good disease-specific survival rate in KIRC and was highly associated with distinct microbes that may have contributed to this outcome. Paradoxically, the receptor was related to the worst prognosis in KIRP with a significant correlation with immune cell infiltration and immunomodulators. Hence, our study outlined a remarkable difference in immune cell subsets and microbiome associated with SUCNR1 in KIRC and KIRP.

Here we first illustrate a decrease in SUCNR1 expression in RCC patients compared to healthy individuals. Even though it was associated with stage IV cancer and the worst prognosis in KIRP patients. The high expression of SUCNR1 in normal kidney tissue might be due to its pivotal function as a physiological sensor, such as maintenance of angiotensin II and renin levels, sodium reabsorption by collecting duct, and proper proximal tubule [43]. Hence, the pathogenic effect is probably related to the accumulation of the metabolite succinate and not to the level of the receptor under pathological conditions. As KIRC possesses a functional deficiency in succinate dehydrogenase, an enzyme complex that oxidizes succinate, due to germline mutation or under-expression, resulting in succinate accumulation [44]. Indeed, the accumulation of succinate has tumorigenic properties exerted through intracellular or extracellular pathways [45].

The succinate–SUCNR1 axis and its downstream signaling are not fully elucidated in cancer [46]. In pheochromocytomas and paragangliomas, SUCNR1 activation is known to induce tumor proliferation [47]. In another study, the receptor was shown to stimulate metastasis in lung cancer and polarized tumor-associated macrophages to the M2 phenotype [48]. Additionally, SUCNR1 was reported with the worst progression-free survival in ovarian cancer [29]. The expression of the receptor was significantly related to cytokine or chemokine gene expression, immune-related gene markers including T cell exhaustion and infiltrating immune cells [29].

Comparably, our data illustrated that high expression of SUCNR1 in KIRP was closely related to lymphocyte infiltration including, activated CD8^+^ T cells, Th1 cells, Th2 cells, γδ T cells, Treg cells, Tfh cells, activated B cells, NK cells, NKT cells, CD56^bright^ NK cells, and CD56^dim^ NK cells. Along with MDSC, activated DC, pDC, mast cells, macrophages, monocytes, and eosinophils. However, even with the association of SUCNR1 with several effector cells such as certain T cells, NK, and DC subsets, the outcome in KIRP was unfavorable. This might be explained by SUCNR1-related regulatory cells (Treg, Th2, MDSC) in the infiltrate [49] and possible desensitization of some effector cells.

Indeed, NK cells and CD8^+^ T cells present in RCC tumors were shown to be non-responsive upon ex vivo stimulation, lack cytolytic activity, granule mobilization, and cytokine production [50,51]. Furthermore, a subset of NK cells was noticed to overexpress CD48, CD85, CD45, and programmed cell death receptor 1 (PD-1) in KIRC [52]. This inhibitory phenotype directly obstructed CD8^+^ T cell proliferation via PD-1 ligand (PD-L1) [53]. Moreover, most of the DC subpopulation in RCC was expressing macrophage markers (CD163, CD14). Thereby reducing chemokine secretion for Th1 cell recruitment, and promoting tumor necrosis factor α by T cells [54]. Mast cell recruitment has also been found to induce RCC angiogenesis in mouse modules [55]. Additionally, MDSC accumulation was accompanied by a negative outcome and a change of inflammatory state in RCC [56]. Regarding macrophages, the population found in tumors were of M1 and M2 phenotypes, secreting IL-6, TNF, and CCL1 [57] and associated with bad prognosis [58].

Our findings also revealed the association of SUCNR1 with fewer infiltrated immune cells including NK cells, eosinophils, mast cells, neutrophils, Th1, Th2, and regulatory T cells in KIRC. This difference in SUCNR1-related immune infiltration between the two RCC subtypes might be due to variability in initial immune cell abundance [30]. Furthermore, immune cell subsets had a distinct impact on survival between both cancers. Whereby, the abundance of Treg cells in KIRC was associated with the worst survival in KIRC, and M2 macrophages showed a bad outcome in KIRP [30].

Our study positively associated SUCNR1 with higher diversity of immune checkpoints in KIRP compared to KIRC. Those immune inhibitors and stimulators are well recognized as pro-tumorigenic. For instance, cytotoxic T-lymphocyte-associated protein 4 (CTLA4) and PD-1 are immune inhibitors that were associated with the worst overall survival in RCC patients [59]. Moreover, CTLA4/miR-20b-5p axis was revealed to induce immune cell infiltration in the KIRC tumor niche [60]. PD-1 ligand, PD-L1, was highly expressed during metastasis in the lung and lymph nodes of the patients. Its expression was related to shorter overall survival [61]. Another PD-1 ligand, PD-L2, was expressed in the tumor microenvironment of RCC and inhibited CD8^+^ T cell activity [62]. To substantiate this further, a different study revealed the association of CTLA4 with poor prognosis in KIRC and PD-L2 in KIRP [30]. One of the immunostimulators, IL-6, was shown essential for the proliferation of cancer cells [63]. CXCR4/CXCL12 axis played an important role in the invasive and migratory phenotype of RCC cells as well [64]. Hence, the potential link of SUCNR1 expression with a pro-tumorigenic immune microenvironment may have explicated the poor prognosis of KIRP patients with high levels of the receptor. Conversely, SUCNR1-related immune cell infiltrates could not unravel its association with a better outcome in KIRC.

There are several probable sources that cause succinate accumulation during pathogenesis including tumor cells, inflammation, and microbiome [21]. Interestingly, extensive studies have associated microbiota dysbiosis with cancer via several methods such as the production of carcinogenic metabolites, modulation of immune response though inflammatory mechanisms, or deregulation of signaling pathways for cell proliferation [65].

Gut microbiota is a major source of succinate and was suggested to induce effects on distant organs including the kidneys [66]. Healthy renal tissue was assumed to be free of microorganisms, even though bacteria can infiltrate the kidneys through the bloodstream [67,68,69]. A recent study described the presence of a plethora of microbiota with a remarkable difference between healthy kidney tissue, and benign and malignant RCC tissue [31]. Consequently, we addressed the correlation of SUCNR1 expression with the present microbiota in RCC subtypes. Indeed, our study further illustrated a difference in microbiome signature in KIRC and KIRP tumor tissues associated with high expression of SUCNR1. The genera *Candidatus nitrosopelagicus* and *Indibacter* belonging to the phyla Thaumarchaeota and Bacteroidetes, respectively, were common in KIRP and KIRC patients with high SUCNR1expression levels.

However, the two bacteria were only related to a better outcome in KIRC patients, especially when SUCNR1 expression is high. Nonetheless, the genera *Anoxybacillus* and *Selenomonas* belonging to the phylum Firmicutes, which were more associated with low SUCNR1 levels, had the worst prognosis in KIRC. As such, the good outcome related to high SUCNR1 expression in KIRC might be related to the presence of beneficial bacteria. This was emphasized by the potential presence of bad bacteria in KIRC tissue in case of receptor under-expression. Additionally, the genus *Apibacter* belonging to the phylum Bacteroidetes was associated with the worst outcome in KIRP when SUCNR1 expression was high. This further explains the relation of SUCNR1 with bad prognosis in KIRP. The majority of bacterial species classified as succinate-producers or succinate-consumers belong to the phyla Bacteroidetes and Firmicutes [66]. In inflammatory bowel disease, changes in serum and fecal succinate levels were related to alterations in succinate-metabolizing bacteria. Accordingly, the succinate-producing bacteria, *B. vulgatus,* was increased in IBD patients. Whereas, the succinate consumer *P. succinatutens* was decreased [70]. Therefore, it might be possible that most of the beneficial microbiota in KIRC are succinate-consumers, unlike KIRP. Our study is the first to describe the presence of these unique bacteria genera in RCC that have a significant effect on the patient’s prognosis. Thus, further examination must be undertaken to classify this microbiota into succinate-producers or -consumers and identify their exact role in cancer. These findings have to be experimentally validated in patient samples and have potential implications on prognostics and therapeutics in RCC.

## 5. Conclusions

In this in silico study we have unraveled SUCNR1 probable function in altering the tumor microenvironment in RCC patients, using correlation analysis of data displayed in public databases. Our study broadens the perception of the SUCNR1 role, for the first time, as not only cell-specific but also as tumor-specific. The findings of this study highlighted the receptor link to immune infiltration and specific microbiome profile. The mechanisms behind this probable divergent role of SUCNR1 in orchestrating immune infiltrates and microbiome population in RCC should be immensely addressed to aid the development of targeted therapies. This study provides the conceptual basis for the involvement of SUCNR1 in the tumor microenvironment, which may be applied to different types of cancers.

## Data Availability

Publicly available datasets were analyzed in this study. This data can be found here: https://www.proteinatlas.org/ (accessed on 1 August 2022), http://cis.hku.hk/TISIDB/ (accessed on 1 August 2022), https://www.cbioportal.org/ (accessed on 1 August 2022), and http://timer.cistrome.org/ (accessed on 1 August 2022).

## Supplementary Materials

The following supporting information can be downloaded at: https://www.mdpi.com/article/10.3390/cancers14246064/s1, Figure S1: Differential SUCNR1 expression among cancers; Figure S2: *Indibacter* and *Candidatus nitrosopelagicus* are beneficial in clear cell RCC; Table S1: SUCNR1 associated microbiome signature in clear cell RCC; Table S2: SUCNR1 associated microbiome signature in papillary RCC; Table S3: Correlation between microbiome and SUCNR1 expression in clear cell RCC; Table S4: Correlation between microbiome and SUCNR1 expression in papillary RCC.

## Abbreviations

KIRC: Kidney Renal Clear Cell Carcinoma, KIRP: Kidney Renal Papillary Cell Carcinoma, Th1: T helper type 1, Th2: T helper type 2, T reg: regulatory T cells, Tfh: T follicular helper cells, γδT: gamma delta T cells, NK: Natural Killer cells, NKT: Natural Killer T cells, DC: Dendritic cells, pDC: plasmacytoid dendritic cells, MDSC: Myeloid-Derived Suppressor cells, CD: Cluster of Differentiation, IL: Interleukin, IL6R: Interleukin 6 Receptor, PDCD1LG2: Programmed Cell Death 1 Ligand 2, PDCD1: Programmed Cell Death Protein, 1TGFB: Transforming Growth Factor Beta, TGFBR: Transforming Growth Factor Beta Receptor, CXCL: chemokine (C-X-C motif) Ligand, CXCR: chemokine (C-X-C motif) Receptor, CSF1R: Colony-Stimulating Factor-1 Receptor, CTLA4: Cytotoxic T-lymphocyte Antigen 4, IDO1: Indoleamine 2,3-Dioxygenase 1, LAG3: Lymphocyte Activating 3, TIGIT: T cell immunoreceptor with Ig and ITIM domains, CD40LG: CD40 Ligand, ICOS: Inducible T Cell Costimulator, ICOSLG: Inducible T Cell Costimulator Ligand, IL2RA: Interleukin 2 Receptor Subunit Alpha.
