# Supplementary material for: Divulging a Pleiotropic Role of Succinate Receptor SUCNR1 in Renal Cell Carcinoma Microenvironment
Source: Cancers (Basel). 2022 Dec 9;14(24):6064. doi: 10.3390/cancers14246064 (PMC9776839; doi:10.3390/cancers14246064)
Supplement: Supplementary file 1 [file cancers-14-06064-s001.zip › cancers-2053218-supplementary.pdf]

Supplementary Materials

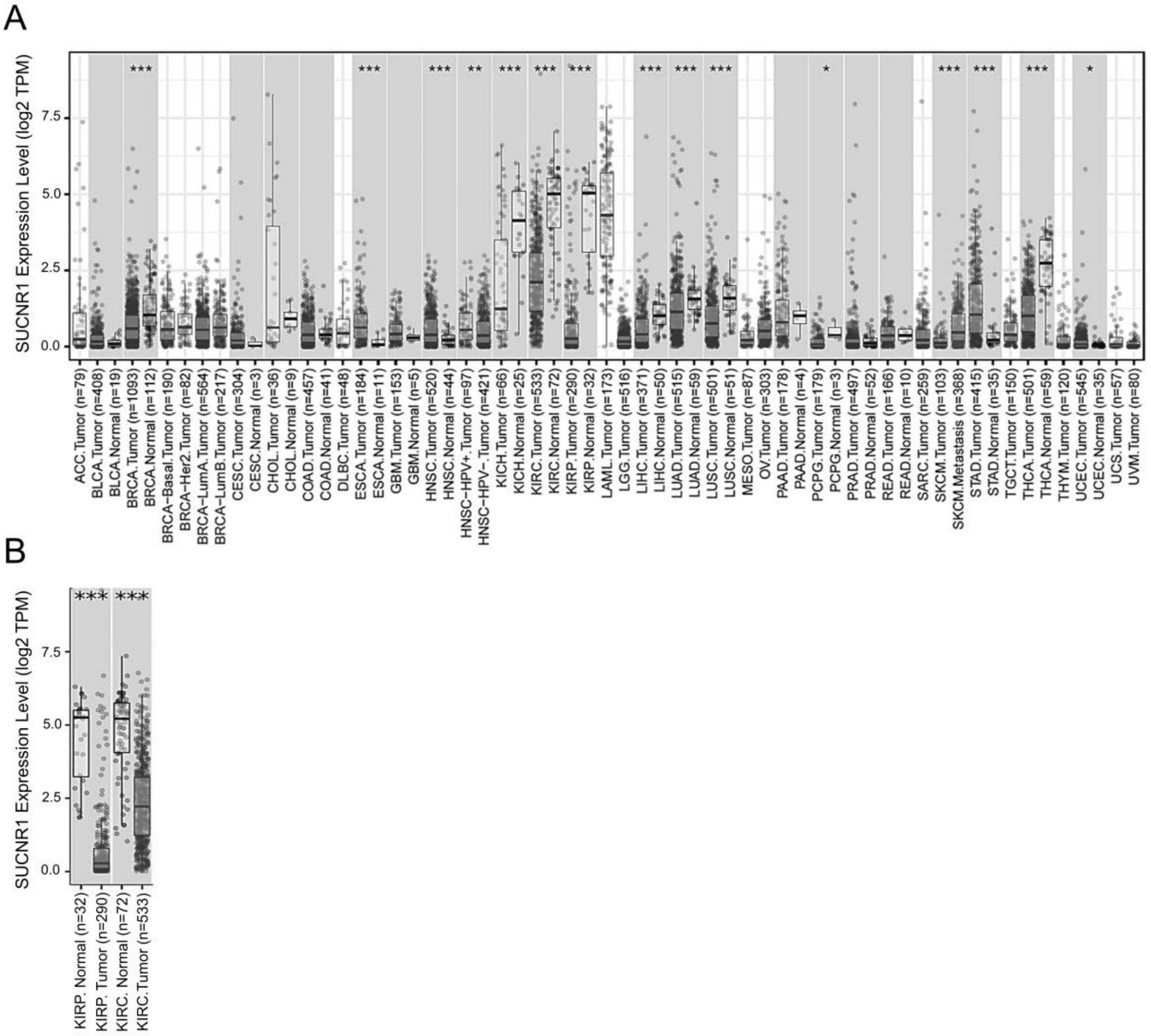

Figure S1. Differential SUCNR1 expression among cancers. (A) Box plots for differential expression of SUCNR1 (Log2 TPM) between normal and tumor tissues of all TCGA tumors. **(B)** Box plots for differential expression of SUCNR1 (Log2 TPM) between normal and tumor tissues of clear cell or papillary RCC. Data available on TIMER2.0 platform. Wilcoxon test; * for *p*-value < 0.05; ** for *p*-value <0.01; *** or **** for *p*-value <0.001.

ACC: Adrenocortical Carcinoma, BLCA: Bladder Urothelial Carcinoma, BRCA: Breast Invasive Carcinoma, CESC: Cervical and Endocervical Cancer, CHOL: Cholangiocarcinoma, COAD: Colon Adenocarcinoma, DLBC: Diffuse Large B-cell Lymphoma, ESCA: Esophageal Carcinoma, GBM: Glioblastoma Multiforme, HNSC: Head and Neck Cancer, KICH: Kidney Chromophobe, KIRC: Kidney Renal Clear Cell Carcinoma, KIRP: Kidney Renal Papillary Cell Carcinoma, LAML: Acute Myeloid Leukemia, LGG: Lower Grade Glioma, LIHC: Liver Hepatocellular Carcinoma, LUAD: Lung Adenocarcinoma, LUSC: Lung Squamous Cell Carcinoma, MESO: Mesothelioma, OV: Ovarian Serous Cystadenocarcinoma, PAAD: Pancreatic Adenocarcinoma, PCPG: Pheochromocytoma and Paraganglioma, PRAD: Prostate Adenocarcinoma, READ: Rectum Adenocarcinoma, SARC: Sarcoma, SKCM: Skin Cutaneous Melanoma, STAD: Stomach Adenocarcinoma, TGCT: Testicular Germ Cell Tumors, THCA: Thyroid Carcinoma, THYM: Thymoma, UCEC: Uterine Corpus Endometrial Carcinoma, UCS: Uterine Carsinosarcoma, UVM: Uveal Melanoma

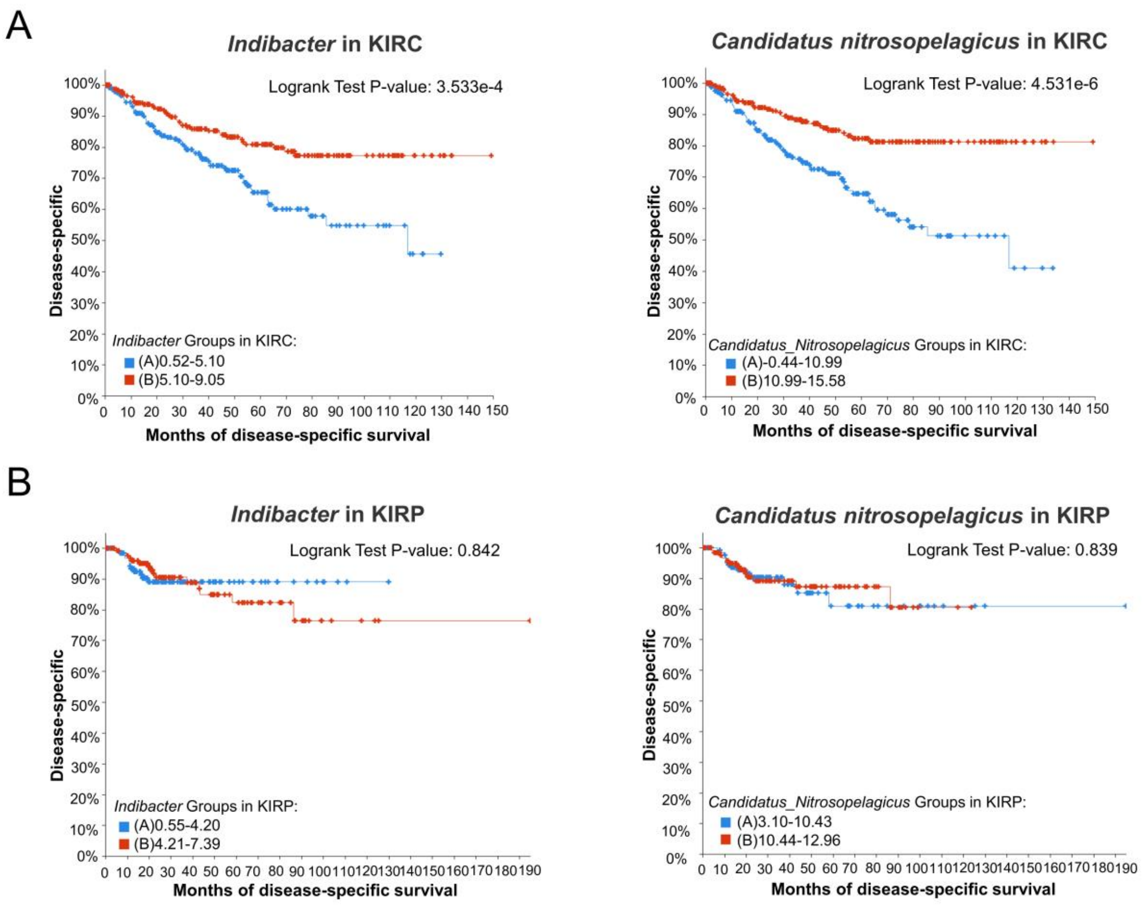

Figure S2. *Indibacter* and *Candidatus nitrosopelagicus* are beneficial in clear cell RCC. (A) Kaplan-Meier curves of low signature group A and high signature group B of *Indibacter* (Logrank test, *p*=3.533e-4) and *Candidatus nitrosopelagicus* (Logrank test, *p*=4.531e-6) in clear cell RCC. **(B)** Kaplan-Meier curves of low signature group A and high signature group B of *Indibacter* (Logrank test, *p*=0.842) and *Candidatus nitrosopelagicus* (Logrank test, *p*=0.839) in papillary RCC. Data available on cBioPortal platform.

Table S1. SUCNR1 associated microbiome signature in clear cell RCC. A table showing the mean of microbiota signature (log CPM) in low expressing SUCNR1 group A and high expressing SUCNR1 group B in clear cell RCC. Data available on cBioPortal platform. Student's t-test *p*-values and Benjamini-Hochberg *q*-values are displayed.

Microbiome Signature	mean in (A)	mean in (B)	p-Value	q-Value	Higher in
Pseudorhodoferax	0.66	0.03	2.69E-04	0.0105	(A)
Azorhizobium	0.88	0.59	3.69E-04	0.014	(A)
Anoxybacillus	0.13	-0.44	4.47E-04	0.0155	(A)
Bacteroides	18.98	18.75	4.48E-04	0.0155	(A)
Altererythrobacter	3.59	3.13	4.66E-04	0.0155	(A)
Draconibacterium	2.05	1.83	6.22E-04	0.0194	(A)

Selenomonas	10.36	10	7.86E-04	0.0226	(A)
Avastrovirus	3.4	2.98	9.92E-04	0.0274	(A)
Isoptericola	1.82	1.47	1.06E-03	0.0288	(A)
Diaphorobacter	-0.41	-0.74	1.14E-03	0.0302	(A)
Alicycliphilus	0.54	-0.02	1.24E-03	0.0323	(A)
Rubrivivax	-0.04	-0.62	1.63E-03	0.0395	(A)
Sediminibacterium	-0.04	-0.53	1.74E-03	0.0414	(A)
Gemmata	13.84	13.72	1.86E-03	0.0435	(A)
Segniliparus	2.38	2.05	1.89E-03	0.0435	(A)
Tannerella	1.46	1.03	1.92E-03	0.0435	(A)
Ramlibacter	4.32	3.85	2.16E-03	0.0475	(A)
Kineococcus	1.15	0.83	2.29E-03	0.048	(A)
Magnetospirillum	4.91	4.66	2.42E-03	0.0492	(A)
Simplexvirus	5.99	6.84	1.72E-14	2.42E-11	(B)
Candidatus nitrosopelagicus	10.36	11.21	1.14E-11	8.01E-09	(B)
Alpharetrovirus	7.56	8.26	2.09E-09	9.80E-07	(B)
Lachnoclostridium	12.24	12.83	7.55E-09	2.65E-06	(B)
Chitinivibrio	8.57	9.4	1.27E-08	3.57E-06	(B)
Maricaulis	3.54	4.24	2.04E-08	4.79E-06	(B)
Campylobacter	16.69	17.17	3.12E-08	6.28E-06	(B)
Indibacter	4.54	5.15	4.54E-08	7.97E-06	(B)
Desulfotalea	5.36	6.03	5.78E-08	9.02E-06	(B)
Aeromonas	16.85	17.33	8.52E-08	1.20E-05	(B)
Riemerella	12.65	12.96	1.57E-07	2.01E-05	(B)
Plantibacter	4.58	5.34	1.79E-07	2.09E-05	(B)
Tunalikevirus	3.98	4.65	2.49E-07	2.69E-05	(B)
Lawsonia	11.12	11.35	4.50E-07	4.52E-05	(B)
Kamptonema	9.09	9.43	4.83E-07	4.53E-05	(B)
Ichnovirus	11.88	12.13	1.72E-06	1.51E-04	(B)
Omegapapillomavirus	4.26	4.91	2.02E-06	1.67E-04	(B)

Lysinimicrobium	3.31	3.85	2.44E-06	1.91E-04	(B)
Bulleidia	3.59	4.11	2.60E-06	1.93E-04	(B)
Mastigocoleus	2.79	3.35	2.79E-06	1.96E-04	(B)
Prevotella	13.51	13.71	3.10E-06	2.07E-04	(B)
Vibrio	15.67	15.89	7.80E-06	4.99E-04	(B)
Coprobacillus	4	4.44	8.72E-06	5.33E-04	(B)
Haliangium	5.54	5.85	1.01E-05	5.94E-04	(B)
Gammaretrovirus	11.54	12.15	1.16E-05	6.52E-04	(B)
Sorangium	9.9	10.19	2.46E-05	1.33E-03	(B)
Microtetraspora	0.04	0.51	2.77E-05	1.44E-03	(B)
Apibacter	3.7	4.05	3.54E-05	1.78E-03	(B)
Entomoplasma	1.66	2.12	5.12E-05	2.48E-03	(B)
Scardovia	2.14	2.72	9.30E-05	4.36E-03	(B)
Alkalibacterium	1.37	1.81	9.79E-05	4.44E-03	(B)
Mannheimia	12.95	13.15	1.21E-04	5.31E-03	(B)
Frankia	5.73	6.05	1.25E-04	5.32E-03	(B)
Treponema	9.88	10.07	2.13E-04	8.82E-03	(B)
Succinimonas	13.09	13.6	2.61E-04	0.0105	(B)
Mycetocola	1.92	2.28	3.95E-04	0.0146	(B)
Rhodobacter	1.52	1.89	4.63E-04	0.0155	(B)
Chromobacterium	4.54	4.94	4.73E-04	0.0155	(B)
Microvirga	13.39	13.61	4.90E-04	0.0157	(B)
Candidatus_Stoquefichus	10.2	10.42	6.73E-04	0.0206	(B)
Ornithobacterium	10.88	11.08	7.71E-04	0.0226	(B)
Gloeobacter	1.96	2.29	7.77E-04	0.0226	(B)
Neorickettsia	5.57	5.99	8.71E-04	0.0245	(B)
Flectobacillus	2.74	3.07	1.32E-03	0.0338	(B)
Flavonifractor	5.04	5.37	1.40E-03	0.0352	(B)
Catenuloplanes	1.7	2.04	1.49E-03	0.0367	(B)
Rhadinovirus	7.66	8.03	2.01E-03	0.0449	(B)

Prymnesiovirus	8.13	8.34	2.23E-03	0.048	(B)
Arenibacter	4.16	4.34	2.26E-03	0.048	(B)
Iflavirus	2.1	2.48	2.36E-03	0.0488	(B)

Table S2. SUCNR1 associated microbiome signature in papillary RCC. A table showing the mean of microbiota signature (log CPM) in low expressing SUCNR1 group A and high expressing SUCNR1 group B in papillary RCC. Data available on cBioPortal platform. Student's t-test *p*-values and Benjamini-Hochberg *q*-values are displayed.

Microbiome Signature	mean in (A)	mean in (B)	p-Value	q-Value	Higher in
Indibacter	3.52	4.26	1.93E-06	2.72E-03	(B)
Candidatus nitrosopelagicus	9.73	10.51	2.13E-05	0.0104	(B)
Lachnoclostridium	11.51	12.21	2.22E-05	0.0104	(B)
Algicola	6.07	6.64	5.94E-05	0.0149	(B)
Apibacter	3.66	4.21	6.20E-05	0.0149	(B)
Desulfotalea	4.87	5.54	6.37E-05	0.0149	(B)
Luteibacter	15.34	15.62	1.41E-04	0.0284	(B)
Paludibacter	8.02	8.59	1.93E-04	0.0338	(B)
Flavonifractor	3.46	4.02	2.31E-04	0.0361	(B)

Table S3. Correlation between microbiome and SUCNR1 expression in clear cell RCC. A table showing the correlation between SUCNR1 expression and microbiome signature that are significanlty present in SUCNR1 expressing groups A and B in clear cell RCC. The correlation between SUCNR1 and microbial expression was analyzed via GraphPad™ software (GraphPad Software, LLC, version 9.4.1) using Spearman's correlation analysis. Statistical significance was reported as following: * for *p*-value < 0.05; ** for *p*-value <0.01; *** or **** for *p*-value <0.001.

Microbiome Signature	Spearman's correlation	p-Value	Higher in
Pseudorhodoferax	-0.1913	****	(A)
Azorhizobium	-0.1425	**	(A)
Anoxybacillus	-0.1905	****	(A)
Bacteroides	-0.1367	**	(A)
Altererythrobacter	-0.1762	****	(A)
Draconibacterium	-0.1171	**	(A)
Selenomonas	-0.1473	***	(A)
Avastrovirus	-0.1511	***	(A)
Isoptericola	-0.1863	****	(A)

Diaphorobacter	-0.1045	*	(A)
Alicycliphilus	-0.1727	****	(A)
Rubrivivax	-0.1736	****	(A)
Sediminibacterium	-0.1376	**	(A)
Gemmata	-0.1525	***	(A)
Segniliparus	-0.1619	***	(A)
Tannerella	-0.1798	****	(A)
Ramlibacter	-0.1798	****	(A)
Kineococcus	-0.1484	***	(A)
Magnetospirillum	-0.205	****	(A)
Simplexvirus	0.3936	****	(B)
Candidatus nitrosopelagicus	0.3033	****	(B)
Alpharetrovirus	0.3146	****	(B)
Lachnoclostridium	0.2419	****	(B)
Chitinivibrio	0.2928	****	(B)
Maricaulis	0.3053	****	(B)
Campylobacter	0.288	****	(B)
Indibacter	0.2673	****	(B)
Desulfotalea	0.2386	****	(B)
Aeromonas	0.2379	****	(B)
Riemerella	0.2881	****	(B)
Plantibacter	0.2145	****	(B)
Tunalikevirus	0.276	****	(B)
Lawsonia	0.2714	****	(B)
Kamptonema	0.2488	****	(B)
Ichnovirus	0.2466	****	(B)
Omegapapillomavirus	0.2099	****	(B)
Lysinimicrobium	0.273	****	(B)
Bulleidia	0.2524	****	(B)
Mastigocoleus	0.2532	****	(B)

Prevotella	0.2354	****	(B)
Vibrio	0.2156	****	(B)
Coprobacillus	0.203	****	(B)
Haliangium	0.2259	****	(B)
Gammaretrovirus	0.2612	****	(B)
Sorangium	0.2127	****	(B)
Microtetraspora	0.1665	***	(B)
Apibacter	0.1749	****	(B)
Entomoplasma	0.2118	****	(B)
Scardovia	0.1357	**	(B)
Alkalibacterium	0.1844	****	(B)
Mannheimia	0.2183	****	(B)
Frankia	0.1745	****	(B)
Treponema	0.1751	****	(B)
Succinimonas	0.1193	**	(B)
Mycetocola	0.1319	**	(B)
Rhodobacter	0.1303	**	(B)
Chromobacterium	0.1396	**	(B)
Microvirga	0.1723	****	(B)
Candidatus_Stoquefichus	0.1624	***	(B)
Ornithobacterium	0.1885	****	(B)
Gloeobacter	0.1293	**	(B)
Neorickettsia	0.1382	**	(B)
Flectobacillus	0.151	***	(B)
Flavonifractor	0.1442	**	(B)
Catenuloplanes	0.1457	***	(B)
Rhadinovirus	0.09689	*	(B)
Prymnesiovirus	0.1501	***	(B)
Arenibacter	0.1368	**	(B)
Iflavirus	0.1755	****	(B)

Table S4. Correlation between microbiome and SUCNR1 expression in papillary RCC. A table showing the correlation between SUCNR1 expression and microbiome signature that are significanlty present in SUCNR1 expressing groups A and B in papillary RCC. The correlation between SUCNR1 and microbial expression was analyzed via GraphPad™ software (GraphPad Software, LLC, version 9.4.1) using Spearman's correlation analysis. Statistical significance was reported as following: * for *p*-value < 0.05; ** for *p*-value <0.01; **** for *p*-value <0.001.

**Microbiome Signature**	**Spearman's correlation**	**p-Value**	**Higher in**
Indibacter	0.2882	****	(B)
Candidatus nitrosopelagicus	0.3492	****	(B)
Lachnoclostridium	0.3437	****	(B)
Algicola	0.2829	****	(B)
Apibacter	0.3012	****	(B)
Desulfotalea	0.309	****	(B)
Luteibacter	0.2455	****	(B)
Paludibacter	0.1281	*	(B)
Flavonifractor	0.176	**	(B)
